# Draft genome sequences of four *Staphylococcus hyicus* strains, SC302, SC304, SC306, and SC310, isolated from swine from Eastern Canada

**DOI:** 10.1128/MRA.00626-23

**Published:** 2023-09-29

**Authors:** Cynthia Gagné-Thivierge, Antony T. Vincent, Valérie E. Paquet, Marie-Lou Gauthier, Martine Denicourt, Marie-Ève Lambert, Steve J. Charette

**Affiliations:** 1 Institut de Biologie Intégrative et des Systèmes (IBIS), Université Laval, Quebec City, Quebec, Canada; 2 Département de Biologie, Faculté des Sciences et de Génie, Université Laval, Quebec City, Quebec, Canada; 3 Département de Biochimie, de Microbiologie et de Bio-informatique, Faculté des Sciences et de Génie, Université Laval, Quebec City, Quebec, Canada; 4 Département Des Sciences Animales, Faculté Des Sciences de L'agriculture et de L'alimentation, Université Laval, Quebec City, Quebec, Canada; 5 Centre de recherche en infectiologie porcine et avicole (CRIPA) - Fonds de recherche du Québec - Nature et technologies, Saint-Hyacinthe, Quebec, Canada; 6 Laboratoire de santé animale, Ministère de l’Agriculture, des Pêcheries et de l’Alimentation du Québec (MAPAQ), Saint-Hyacinthe, Quebec, Canada; 7 Département de sciences cliniques, Faculté de médecine vétérinaire, Université de Montréa, Saint-Hyacinthe, Quebec, Canada; Wellesley College Department of Biological Sciences, Wellesley, Massachusetts, USA

**Keywords:** *Staphylococcus hyicus*, tetracycline, exudative epidermitis, piglet, Eastern Canada, phylogeny

## Abstract

The bacterium *Staphylococcus hyicus* causes porcine exudative epidermitis in piglets, which represents both health and welfare concerns. Few genome sequences of this pathogen are published. We provide four additional ones to help future genomic analysis of *S. hyicus*. These are genomes of strains isolated from Canadian swine.

## ANNOUNCEMENT

*Staphylococcus hyicus* is the causative agent of porcine exudative epidermitis (EE) in piglets, which affects swine farms sporadically, as *S. hyicus* is endemic in most herds worldwide (1). Despite the problems caused, few *S. hyicus* genome sequences are currently available. We aim to better understand *S. hyicus* by increasing available genome sequences.

We present draft genome sequences of four *S*. *hyicus* strains isolated from Eastern Canada. The strains (SC302, SC304, SC306, and SC310) were isolated and initially identified by the Ministère de l’Agriculture, des Pêcheries et de l’Alimentation du Québec (MAPAQ) veterinary diagnostic services in 2021, following necropsy of piglets submitted for clinical signs of EE and/or lameness. Tissue samples (Table 1) were plated onto 5% sheep blood agar, incubated at 35°C in 5% CO_2_. Colonies selected based on morphology were identified using MALDI-TOF mass spectrometry (MALDI biotyper smart, Bruker Daltonics, Bremen, Germany). Confirmed *S. hyicus* colonies were streaked onto blood agar to ensure purity before being inoculated into tryptic soy broth (TSB) + 20% glycerol and frozen at −80°C.

**TABLE 1:** Sampling information as well as sequencing and assembly statistics for the strains presented in this study

Strain	Veterinary diagnosis	Sampled tissue	Assembly size (bp)	Number of contigs	N_50_ value (bp)	GC content (%)	Number of reads	Coverage (×)	Genome accession number	SRA accession number
SC302	Lameness	Navel lesion	2,488,674	50	276,159	35.46	1,983,118	165	JAUBYW000000000	SRR25222503
SC304	EE	Skin lesion	2,545,127	45	277,961	35.51	826,828	72	JAUBYX000000000	SRR25222502
SC306	EE + lameness	Skin lesion, kidney, or tarsus	2,462,965	44	599,968	35.57	1,036,134	89	JAUBYY000000000	SRR25222501
SC310	EE	Skin lesion	2,571,865	68	166,310	35.44	1,257,356	98	JAUBYZ000000000	SRR25222500

After recovery from frozen stocks, plating on TSA, and incubation at 37°C for 24 hours, an inoculum of each strain was grown in TSB at 37°C, 200 rpm, for subsequent genomic DNA extraction using DNeasy Blood and Tissue Kits (Qiagen, Canada), according to the manufacturer’s instructions. The purified DNA was prepared into sequencing libraries using NEBNext Ultra II FS DNA Library Prep Kits (Ipswich, MA, USA) and sequenced using a MiSeq instrument system (Illumina, San Diego, CA, USA) generating 2 × 300 bp reads (Plateforme d’Analyses Génomiques, IBIS, Université Laval). Sequencing reads were verified with FastQC version 0.11.8 (2), filtered using fastp version 0.23.2 (3), and *de novo* assembled in contigs using Shovill version 1.1.0 (4). This Whole-Genome Shotgun project has been deposited at DDBJ/EMBL/GenBank under BioProject PRJNA985240. Default parameters were used for all bioinformatics tools, unless otherwise specified. Assembly statistics are presented in Table 1.

**Fig 1:**
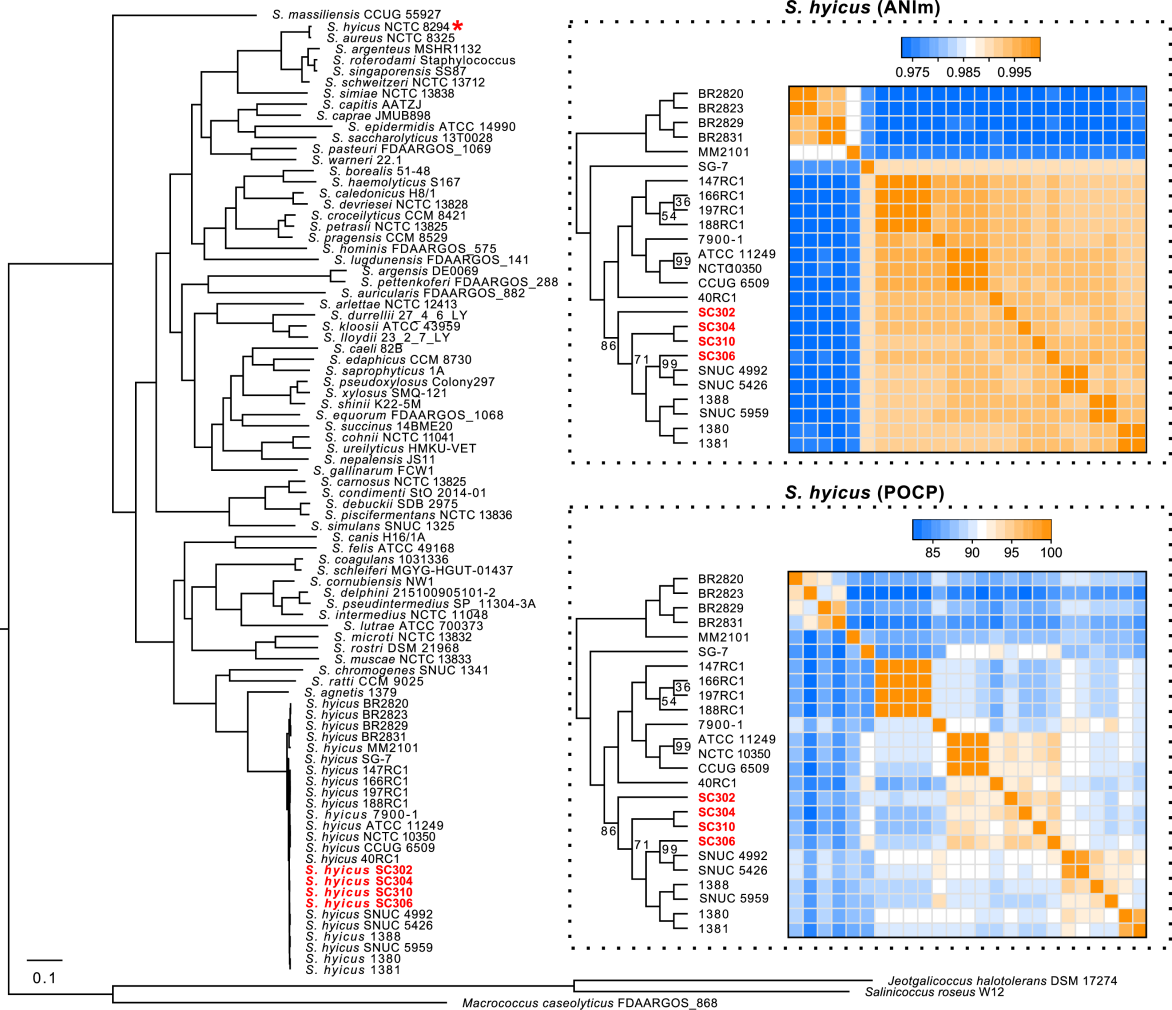
Molecular phylogeny of the genus *Staphylococcus* with emphasis on species *hyicus*. Orthologous genes were identified with COG and OMCL through GET_HOMOLOGUES version 20220822 (5). The sequences of the 1,003 orthologous gene clusters were aligned with mafft version 7.515 (6) and TranslatorX and finally filtered with BMGE version 1.12 (7). The phylogenetic tree was then made with IQ-TREE version 2.2.0 (8), with 10,000 ultrafast bootstraps, choosing the best model for each of the 1,003 partitions. The four strains of *S. hyicus* whose genomes were sequenced by the present study are in red. The ANIm matrix allows evaluating the nucleotide diversity, and the POCP values are based on the orthologous proteins shared between the strains. The values of ANIm and POCP were computed using pyani version 0.2.12 (9) and GET_HOMOLOGUES version 22082022 (5), respectively. Bootstrap values are shown when less than 100. Strain NCTC 8294 (BioSample number SAMEA3499053) was revealed to be a *S. aureus* strain (red asterisk) in the phylogeny which explains its exclusion from ANIm and POCP analysis.

Taxonomic identification was performed by molecular phylogeny (Fig. 1) including the 22 *S*. *hyicus* genome sequences currently available on GenBank, 1 reference strain for each of 64 other Staphylococci species, and 3 closely related genera as outgroup. The phylogeny was coupled for *S. hyicus* strains with average nucleotide identity (ANIm) analysis and calculation of a matrix of the percentage of conserved proteins (POCP) values. POCP results are especially useful to show distinctiveness of strains.

The four genomes presented are from Eastern Canada, where swine industry is important. Interestingly, POCP results suggest diversity in these strains despite being from the same region and with other Canadian strains isolated from cattle (strains SNUC XXXX, Fig. 1). Strains CCUG 6509, NCTC 10350, and ATCC 11249 regroup as they come from the same original isolate (10–12). Another group forms between isolates from same BioProject (strains XXXRC1), suggesting clonality or close link.

Sequencing these strains was partly aimed to analyze their possible resistance to tetracycline, which is largely used in swine industry (13). We found two distinct tetracycline resistance genes: SC304 possesses a chromosomal *tet*(M) gene on a Tn916/Tn1545 transposon, whereas SC310 possesses a *tet*(L) gene on an unknown plasmid.

## Data Availability

The genome sequences of the four *Staphylococcus hyicus* strains have been deposited in DDBJ/ENA/GenBank under the following accession and BioSample numbers: JAUBYW000000000 and SAMN35793890 for SC302, JAUBYX000000000 and SAMN35793891 for SC304, JAUBYY000000000 and SAMN35793892 for SC306, and JAUBYZ000000000 and SAMN35793893 for SC310.
